# Zucchini Yellow Mosaic Virus Infection Limits Establishment and Severity of Powdery Mildew in Wild Populations of *Cucurbita pepo*

**DOI:** 10.3389/fpls.2018.00792

**Published:** 2018-06-13

**Authors:** Jacquelyn E. Harth, Matthew J. Ferrari, Anjel M. Helms, John F. Tooker, Andrew G. Stephenson

**Affiliations:** ^1^Department of Biology, The Pennsylvania State University, University Park, PA, United States; ^2^Center for Infectious Disease Dynamics, The Pennsylvania State University, University Park, PA, United States; ^3^Department of Entomology, The Pennsylvania State University, University Park, PA, United States

**Keywords:** *Cucurbita pepo*, multi-parasite interactions, powdery mildew, systemic acquired resistance, ZYMV

## Abstract

Few studies have examined the combined effect of multiple parasites on host fitness. Previous work in the *Cucurbita pepo* pathosystem indicates that infection with Zucchini yellow mosaic virus (ZYMV) reduces exposure to a second insect-vectored parasite (*Erwinia tracheiphila*). In this study, we performed two large-scale field experiments employing wild gourds (*Cucurbita pepo* ssp. *texana*), including plants with a highly introgressed transgene conferring resistance to ZYMV, to examine the interaction of ZYMV and powdery mildew, a common fungal disease. We found that ZYMV-infected plants are more resistant to powdery mildew (i.e., less likely to experience powdery mildew infection and when infected with powdery mildew, have reduced severity of powdery mildew symptoms). As a consequence, during widespread viral epidemics, proportionally more transgenic plants get powdery mildew than non-transgenic plants, potentially mitigating the benefits of the transgene. A greenhouse study using ZYMV-inoculated and non-inoculated controls (non-transgenic plants) revealed that ZYMV-infected plants were more resistant to powdery mildew than controls, suggesting that the transgene itself had no direct effect on the powdery mildew resistance in our field study. Additionally, we found evidence of elevated levels of salicylic acid, a phytohormone that mediates anti-pathogen defenses, in ZYMV-infected plants, suggesting that viral infection induces a plant immune response (systemic acquired resistance), thereby reducing plant susceptibility to powdery mildew infection.

## Introduction

Many studies have examined the costs of parasite infection for host fitness. In both plant and animal systems, parasites can increase mortality, reduce reproductive output, reduce growth, and induce defense systems that are costly in terms of energy and nutrients (e.g., Anderson and May, 1978; Burdon and Leather, 1990; Ryals et al., 1996; Hawkins et al., 1997; Maskell et al., 1999; Moore, 2002a,b). The presence of multiple parasites in a host, which is common in nature, can change the expected dynamics of these systems (Petney and Andrews, 1998; Cox, 2001; Lello et al., 2004; Hatcher et al., 2006; Lively et al., 2014). Despite the complex nature of most natural communities, relatively few empirical studies have examined the combined effect of multiple parasites on host fitness especially under field conditions (e.g., Mitchell and Power, 2006; Pedersen and Fenton, 2006; Sasu et al., 2009; Alexander, 2010).

Direct interactions, such as interference competition between parasites, have been well documented (Dobson and Barnes, 1995). However, the importance of indirect effects as drivers of parasite community structure are gaining attention (Strauss, 1991; Pedersen and Fenton, 2006). Indirect interactions among parasites can influence parasite fitness (Stewart et al., 1999; Ishii et al., 2002), host fitness (Donoghue et al., 2005), and have important implications for the spread of parasites within communities (Mitchell and Power, 2006). These indirect interactions fall into two broad categories: pre-infection interactions (i.e., interactions that affect host exposure to a second parasite) and post-infection interactions (i.e., infection with one parasite changing host resource quality or the host immune response to a second parasite).

Pre-infection interactions among competing parasites have been documented in a few study systems. “Ecological interference” has been proposed as a mechanism of interaction in directly transmitted childhood infections, whereby illness due to a primary infection reduces contact rates and therefore exposure to a secondary infection (Rohani et al., 1998, 2003). Vector behavior, in response to an infected host, can also influence exposure rates to a vectored parasite. Changes in host-plant chemistry after infection can alter volatile cues that then influence vector-feeding preferences (Mauck et al., 2010). These changes in vector behavior can result in either increased (Wang et al., 2014) or reduced exposure of the host to a secondary parasite (Shapiro et al., 2012).

After infection, a co-infecting parasite enters an environment in which host resources may already be consumed by an initial parasite, or in which host defenses are upregulated in response to the primary infection. In plant cells, infection can trigger signaling cascades of the innate immune system that provide broad spectrum immunity against subsequent parasite attack (Jones and Dangl, 2006). These signaling cascades function via the signaling molecules, jasmonic acid (JA) and salicylic acid (SA), which are key mediators of plant defense responses. Specifically, SA is considered the primary phytohormone mediating induced resistance against biotrophic pathogens (An and Mou, 2011), while JA is primarily responsible for regulating defenses against necrotrophic pathogens and herbivores (Wasternack, 2007). In particular, SA is essential to the plant immune system, facilitating broad spectrum protection provided by systemic acquired resistance (SAR), cell-wall fortification, and the accumulation of pathogenesis related (PR) proteins (Dean and Kuc, 1987; Vlot et al., 2009; An and Mou, 2011). A SAR response triggered by the presence of one parasite has been shown to increase defenses against subsequent parasite attack in a few systems (Kuc, 1982; Mucharromah and Kuc, 1991). Additionally, antagonistic “cross-talk” between the SA and JA pathways can also influence the interaction among multiple parasites in the same host (Kunkel and Brooks, 2002; Chung et al., 2013).

We have extensively studied the interactions among pathogens and herbivores in populations of *Cucurbita pepo* ssp. *texana* (e.g., Hayes et al., 2004; Stephenson et al., 2004; Ferrari et al., 2007; Du et al., 2008; Sasu et al., 2009, 2010a,b; Simmons et al., 2011; Shapiro et al., 2012, 2014; Harth et al., 2016) under laboratory, greenhouse and field conditions. *Cucurbita pepo* ssp. *texana* (wild gourd) is an annual monoecious vine that is cross compatible with cultivated squash and several annual species of wild *Cucurbita* (Decker-Walters et al., 1993; Arriaga et al., 2006). The primary herbivores of wild gourds include a variety of generalist aphids and several species of cucumber beetles. The aphids also vector the four most common viral diseases of cucurbits [Cucumber mosaic (CMV), Papaya ringspot, Watermelon mosaic (WMV), and Zucchini yellow mosaic virus (ZYMV)]. ZYMV and WMV are the two most common viral diseases of cucurbits at our field sites in Central Pennsylvania (Sasu et al., 2009). Both viruses are in the family Potyviridae and are transmitted via aphids in a non-persistent manner. These viruses depress reproductive output but they do not typically kill the plants (e.g., Stephenson et al., 2004). Cucumber beetles are the only known vector of *Erwinia tracheiphila*, the causative agent of bacterial wilt disease. Transmission occurs when fecal pellets containing *Erwinia* fall either onto open wounds created during feeding or onto the floral nectaries when beetles aggregate in the flowers to mate (Fleischer et al., 1999; Sasu et al., 2010b). Wilting symptoms develop 10–15 days after infection and the disease is fatal once symptoms appear (Ferrari et al., 2007).

At our field sites in Central Pennsylvania, the *C. pepo* pathosystem also consists of powdery mildew (*Podosphaera* sp.), a wind-dispersed fungal pathogen of squash, cucumbers and melons. The fungus overwinters as chasmothecia (sexual spores) which serve as the source of initial infection each year. However, conidia (asexual spores) are the primary means of dispersal. Symptoms first appear on older leaves as small reddish-brown spots that quickly turn white as hyphae are produced. As the disease progresses, leaves become covered in the powdery, white mycelia before shriveling and dying. In central Pennsylvania, first symptoms typically occur in early August and by September the disease will spread to the entire population.

We have previously documented that primary infection with ZYMV limits exposure to *Erwinia tracheiphila*, i.e., a pre-infection interaction, by changing the foraging behavior of the beetle vector (Sasu et al., 2009, 2010a; Shapiro et al., 2012). These studies were greatly aided by the fact that in 1996, the USDA deregulated a transgenic yellow crookneck squash (Asgrow, CZW-3) with coat protein-based resistance to WMV, ZYMV, and CMV (USDA, 1996). In short, the transgene consists of a constitutively expressed promoter that drives the expression of portions of the coat protein of each of the three viruses which act as interference RNA to prevent encapsulation during viral replication (Fuchs and Gonsalves, 2007). By introgressing the transgene into wild gourd, we were able to create plants that were susceptible only to bacterial wilt disease and plants that were susceptible to ZYMV and bacterial wilt. In the study presented below, we show using large scale field and greenhouse experiments that a primary infection with ZYMV increases SA production and has post-infection effects on the incidence and severity of powdery mildew in populations of *C. pepo* ssp. *texana*.

## Materials and Methods

### Introgression of the Virus Resistant Transgene and Field Experimental Design

To examine the effects of ZYMV infection on subsequent powdery mildew infection and establishment in wild gourds, under field conditions, we used transgenic wild gourds that were resistant to ZYMV but susceptible to powdery mildew and wild gourds that were susceptible to both ZYMV and powdery mildew. The Liberator III crookneck squash cultivar is one of many commercially available transgenic squash with coat protein based resistance to WMV, ZYMV, and CMV. In this cultivar, the virus resistance transgene (hereafter called the transgene) is hemizygous and, importantly, the *NPTII* marker gene has not been deactivated and is still tightly linked to the coat protein genes of the three viruses. Consequently, we have been able to introgress the transgene (coat protein genes and *NPTII*) into 20 families of the wild gourd (using the wild gourd as the recurrent parent) because the presence of the NPTII protein in hybrid progeny can be detected serologically. Previous studies have shown that the introgressed transgene effectively deters the target viruses under field conditions (Sasu et al., 2009, 2010a). For the study reported here, we used non-transgenic (ntBC9) and transgenic plants (tBC9) from the backcross nine generation (BC9) as well as wild type wild gourds. The BC9 plants are expected, on average, to possess less than 0.1% of the nuclear DNA of the Liberator III cultivar and no organelle DNA from the cultivar. It was not possible for us to distinguish among the three types of plants (wild gourds, ntBC9, tBC9) based upon appearance or growth habit. Moreover, because we screened progeny from multiple tBC8 X wild gourd crosses to produce the tBC9 and ntBC9 plants used in this study, we know that the segregation of the transgene did not differ significantly from 50 to 50 (see Sasu et al., 2009 for similar results from previous generations).

We germinated seeds in a greenhouse in early May 2013 and 2014 and, in late May of each year, we transplanted 18 wild gourds (wild type ssp. *texana*) plants, 9 tBC9, and 9 ntBC9 from each of five families (180 total plants per field, 25% were transgenic) into each of four 0.4 ha fields at the Pennsylvania State University Agriculture Experiment Station at Rock Springs, PA. In two of the four fields (located 25 m apart) we inoculated eight wild gourd and four ntBC9 from each of the five families prior to transplanting. Inoculum was prepared by grinding 2–3 ZYMV infected symptomatic leaves in 100 ml of a phosphate buffer. Plants were then inoculated by brushing the adaxial leaf surface with 320 grit carborundum and then dipping a small pestle into the inoculum and gently rubbing the leaf surface in a circular motion. This inoculation design resulted in 60 inoculated plants, 75 non-inoculated wild gourds and ntBC9 (virus susceptible) plants, and 45 tBC9 plants per field (**Supplementary Figure S1**). Symptoms first appeared 7–10 days after inoculation. The other two fields had no inoculated plants and were located approximately 1 km from the fields with the inoculated plants. The non-inoculated fields were also separated by 25 m and they were permitted to become naturally infected with mosaic viruses. In all four fields, the plants were the progeny from the same five randomly selected families (by family we mean the wild gourd, ntBC9, and tBC9 progeny shared the same maternal parent). Moreover, the tBC9 and ntBC9 from each family were full siblings (i.e., sired by the pollen from one hemizygous tBC8 plant from one of five different families) as were all of the wild gourd plants from a given maternal parent. In all four fields, plants were allowed to become infected with powdery mildew naturally.

The plants in each of the four fields were monitored once weekly throughout the growing season (beginning in early June) for symptoms of viral diseases. Our field diagnoses based upon visual symptoms were confirmed in both early July and early August by taking leaf samples from up to 20 symptomatic and 20 asymptotic plants per field and testing the leaves using ZYMV ImmunoStrips^®^ (purchased from Agdia Inc., Elkhart, IN, United States. No inoculated plants were tested and no symptomatic plants were tested in both July and August. In the two control fields (no inoculations of ZYMV) it was not possible to find 20 symptomatic plants in early July. In no case did our field diagnosis differ from the immunological test results. However, it should be noted that the ELISA based ImmunoStrips are not as sensitive as RT-PCR screening. Consequently, it possible that some asymptomatic plants had low titers of ZYMV (Prendeville et al., 2012) and were pre-symptomatic. During our weekly screening for ZYMV symptoms we also screened for symptoms of powdery mildew. In mid-August of each year, we collected a sample of infected leaves from 15 visually infected plants in each field and our field diagnosis was confirmed by the Plant Disease Clinic, Pennsylvania State University, University Park, PA, United States, as *Podosphaera* sp. (most likely *Podosphaera xanthii*). We assessed powdery mildew symptom severity on a 0–3 scale where 0 indicates a plant showing no powdery mildew symptoms, 1 indicates a plant with <33% of leaves showing >50% spread of powdery mildew symptoms on the leaf surface, 2 indicates a plant with 33–67% of leaves showing >50% spread of powdery mildew symptoms on the leaf surface, and 3 indicates a plant with >67% leaves showing >50% spread of powdery mildew symptoms on the leaf surface. We assessed reproductive output by counting staminate (male) and pistillate (female) flowers weekly (an unbiased estimate of total flower production because the flowers last for only one morning) until the beginning of September.

### Greenhouse Experimental Design

To confirm that the interaction between ZYMV and powdery mildew was not a direct result of transgene presence, we grew 320 wild gourd plants (wild type, no transgenic plants) in a greenhouse in September 2014. Additionally, the greenhouse environment allowed for a controlled environment where the only difference between plants prior to powdery mildew introduction was presence or absence of ZYMV. We inoculated 160 plants (16 plants from each of 10 families) with ZYMV at the second true leaf stage using the procedure described above (ZYMV infection confirmed by ELISA based ImmunoStrips; Agdia Inc., Elkhart, IN, United States). We mock-inoculated 80 (8 plants from each of 10 families) plants using just the inoculation buffer and left 80 plants as untreated controls (8 plants from each of 10 families). Plants were arranged in a randomized block design. Plants were treated with an insecticide to prevent leaf herbivory and ZYMV spread. Fourteen days after all inoculated plants first showed ZYMV symptoms, 12 additional plants with established powdery mildew infections were moved into the greenhouse and uniformly distributed. Fans were used to circulate the spores and ensure all plants were rapidly exposed to the powdery mildew inoculum. Plants were scored for powdery mildew symptoms every 2 days for 3 weeks.

To examine the effects of ZYMV infection on SA concentration in leaves, we grew an additional 12 plants for each treatment (mock, ZYMV-infected, and control) to use for phytohormone analysis. Leaf samples weighing 0.1 g were collected from these plants 10 days post-inoculation (prior to the introduction of powdery mildew) and were taken from new leaves that were systemically infected. Samples were immediately flash frozen in liquid nitrogen. After harvesting, the tissue samples received 100 ng of internal, isotopic standards and 400 μL of an aqueous, weakly acidic buffer solution while on ice. After grinding and mixing, each sample then received 1 mL of dichloromethane and was mixed and centrifuged for 2 min at 12,000 × *g*. The dichloromethane layer, now containing the compounds of interest and the internal standards, was removed to a glass vial and dried under house air. Briefly, after solvent extractions of leaf tissue we derivatized SA to its methyl ester, which we isolated using vapor-phase extraction and analyzed by coupled gas chromatography-mass spectrometry with isobutane chemical ionization using selected-ion monitoring. We quantified amounts of total free SA using 100 ng of [^2^H_6_]SA (CDN Isotopes, Pointe-Claire, Montreal, QC, Canada) (Schmelz et al., 2003, 2004; Shapiro et al., 2013).

## Results

### Effect of ZYMV on Powdery Mildew Spread and Establishment – Field Experiments

Our initial analyses revealed that the wild type gourds and the ntBC9 plants did not differ statistically in their incidence or severity of powdery mildew infection, nor in their flower production. Therefore, we combined these two plant types into a single “susceptible’ class for the analyses presented below. Plants that died before the first incidence of powdery mildew in our fields (from transplant stress and bacterial wilt disease) were not included in these analyses.

**FIGURE 1:**
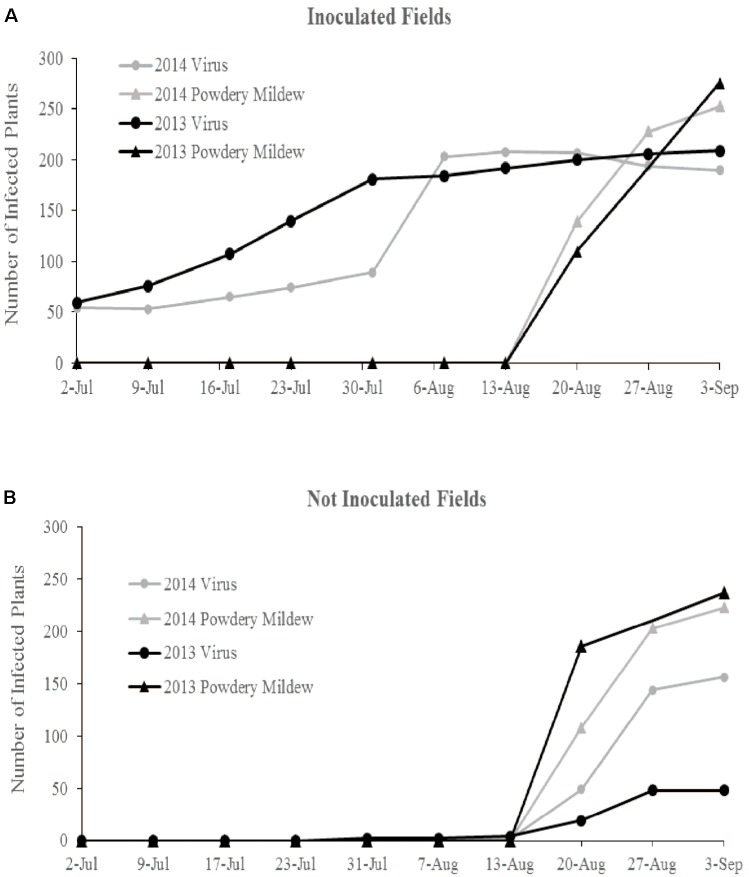
Cumulative total of ZYMV infected plants and plants with powdery mildew symptoms over the course of both field seasons in **(A)** the ZYMV inoculated fields and **(B)** the not inoculated fields.

In 2013, flowering began in late June and peak flower and fruit production occurred in August. Powdery mildew first appeared in mid-August and spread rapidly in all fields. Natural ZYMV spread in the inoculated fields began in mid-June, with peak spread occurring before first powdery mildew incidence (**Figure 1A**). In the non-inoculated fields, first ZYMV incidence occurred in late July. The transgene was effective against ZYMV infection, though a few transgenic plants did express mild symptoms ZYMV infection in August (**Supplementary Table S1**).

**FIGURE 2:**
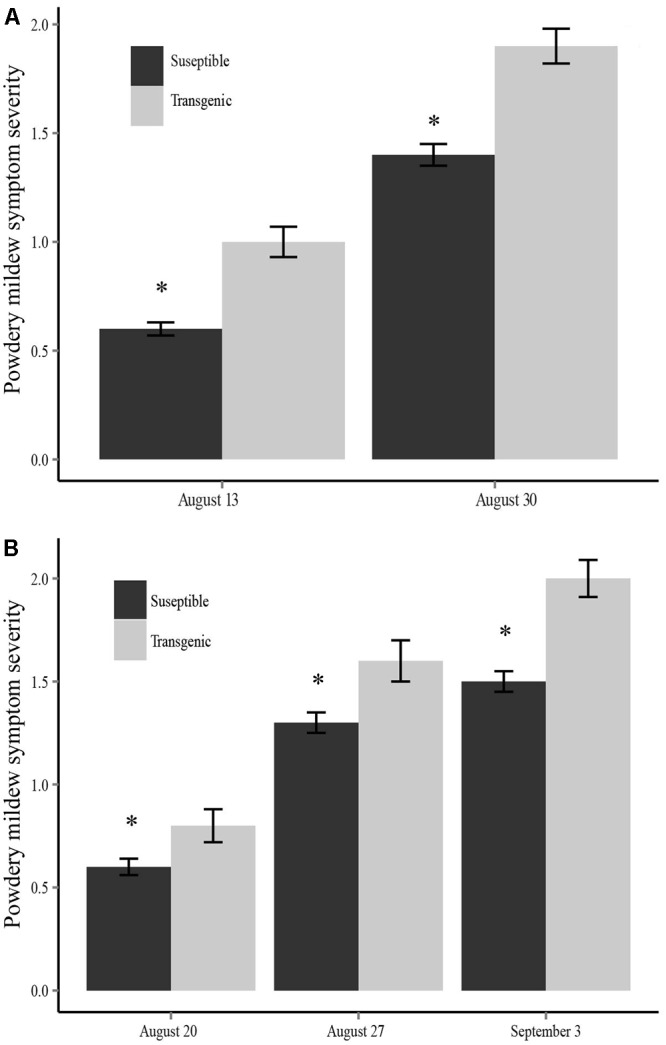
Powdery mildew symptom severity for each sampling date comparing transgenic and susceptible plants in **(A)** the 2013 field season and **(B)** the 2014 field season. Transgenic plants had significantly more severe symptoms at each time point (^∗^*P* < 0.05).

In 2013, there were significant differences in the incidence of powdery mildew between the two plant types (susceptible and transgenic) in both the inoculated fields (*X*^2^ = 23.173, *df* = 1, *P* < 0.001) and the non-inoculated fields (*X*^2^ = 6.876, *df* = 1, *P* = 0.009) in mid-August, with the same trend continuing in late August. We used a mixed-effects analysis of variance (ANOVA) to assess the effect of plant type (susceptible or transgenic), family (random), treatment (virus inoculated fields or no inoculation fields), and a plant type by treatment interaction on powdery mildew symptom severity. We found that transgenic plants not only had a higher incidence of powdery mildew infection, they also experienced significantly more severe powdery mildew symptoms than virus susceptible plants at each time point during the 2013 field season (mid-August: *F*_1,564_ = 34.10, *P* < 0.001; late August: *F*_1,564_ = 29.30, *P* < 0.001) (**Figure 2A**). A mixed-effects model ANOVA showed there was not a significant plant-type by treatment interaction (*F*_1,564_ = 2.23, *P* = 0.078) for the final powdery mildew scoring date (late August). However, inoculation treatment was not a significant factor indicating that ZYMV spread in the non-inoculated fields had reached a point where there were no longer treatment (field inoculation) differences. Additionally, there were significant family differences for powdery mildew severity at both time points (mid-August: *F*_4,564_ = 3.94, *P* < 0.01; late August: *F*_4,564_ = 6.07, *P* < 0.001), with one family showing the most severe symptoms (**Supplementary Figure S2A**).

In 2014, flowering began in late June and peak flower production occurred in August. Powdery mildew first appeared in mid-August and spread rapidly in all fields. In the inoculated fields, natural ZYMV spread began in late June and peaked in late July, with most susceptible plants (wild gourd and ntBC9) infected before first incidence of powdery mildew. In the non-inoculated fields, first incidence of ZYMV was in mid-August, occurring simultaneously with powdery mildew infection and spreading rapidly (**Figure 1B**). The transgene was effective against ZYMV infection, though a few transgenic plants did express mild symptoms of virus in August.

In mid-August 2014, the incidence of powdery mildew was significantly higher on transgenic plants in the virus inoculated fields (*X*^2^ = 21.368, *df* = 1, *P* < 0.001) than on the susceptible plants. However, there was no significant difference in the incidence of powdery mildew on transgenic and susceptible plants in the non-inoculated fields (*X*^2^ = 0.068, *df* = 1, *P* = 0.794) indicating that the transgene *per se* does not alter the resistance of plants to powdery mildew. However, by early September, after virus had spread through the non-inoculated fields, there were significant differences in the proportion of transgenic and susceptible plants that contracted powdery mildew (*X*^2^ = 4.982, *df* = 1, *P* = 0.026).

We found that transgenic plants not only had a higher incidence of powdery mildew infection, they also experienced significantly more severe powdery mildew symptoms than non-transgenic plants at each time point during the 2014 field season (mid-August: *F*_1,522_ = 7.06, *P* < 0.01; late August: *F*_1,522_ = 6.65, *P* < 0.05; early September: *F*_1,522_ = 16.92, *P* < 0.001) (**Figure 2B**). The ANOVA also revealed a significant positive plant-type by treatment (inoculated vs. non-inoculated fields) interaction term indicating that transgenic plants have more severe powdery mildew symptoms in the ZYMV-inoculated fields (mid-August: *F*_1,522_ = 9.27, *P* < 0.01; late August: *F*_1,522_ = 4.13, *P* < 0.05; early September: *F*_1,522_ = 4.35, *P* < 0.05). Additionally, there were significant family differences for powdery mildew severity (mid-August: *F*_4,522_ = 2.80, *P* < 0.05; late August: *F*_4,522_ = 2.76, *P* < 0.05; early September: *F*_4,522_ = 2.65, *P* < 0.05), with one family showing the most severe symptoms (**Supplementary Figure S2B**).

Among susceptible plants in our fields, the proportion of plants that contracted powdery mildew during the initial outbreak decreased with the number of weeks they were infected with ZYMV (0–10 weeks) (**Supplementary Figure S3**). Moreover, transgenic and susceptible plants that were not virus infected had a similar incidence of powdery mildew during the initial stages of the powdery mildew outbreak (**Supplementary Figure S3**). We used a logistic regression model to assess the effect of plant type (transgenic or susceptible), family (random), treatment (inoculated or non-inoculated fields), and ‘weeks with virus symptoms,’ on the likelihood of infection with powdery mildew. This analysis showed that weeks with virus is a significant predictor of powdery mildew incidence and for each week a plant has been infected with virus, ZYMV-infected plants are 0.84 times less likely to contract powdery mildew. A reduced model that did not include weeks with virus showed that when powdery mildew first enters our fields, the odds of a transgenic plant contracting powdery mildew are 2.4 times that of susceptible plants (most of which are infected with ZYMV at this time in the season). However, the full model is a better fit (AIC full model = 1459.6; AIC reduced model = 1497.5) and ‘when weeks with virus’ is included in the model, plant type is no longer a significant predictor. This suggests that differences between transgenic and susceptible plants are a result of viral infection and not the transgene *per se*. The logistic regression model also revealed a significant family effect. In particular, one maternal family showed higher odds of contracting powdery mildew relative to the other four families suggesting that there was heritable (in the broad sense) variation for resistance to powdery mildew among families.

### Effect of ZYMV on Powdery Mildew Spread and Establishment – Greenhouse Experiment

**FIGURE 3:**
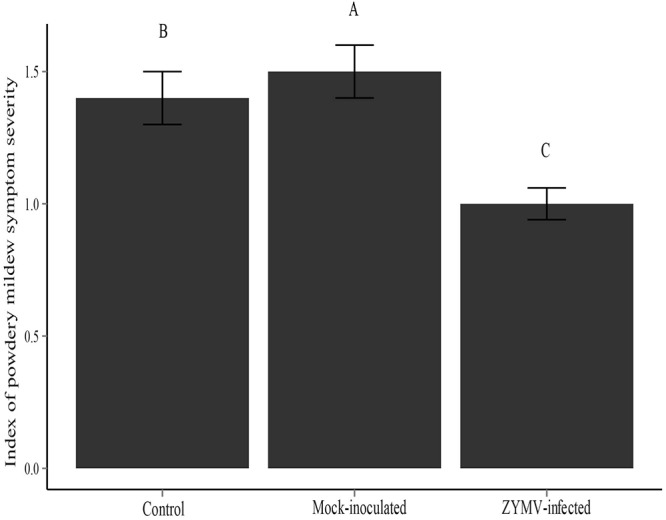
Severity of powdery mildew symptoms for each treatment type for the greenhouse experiment. Different letters indicate significant differences between groups at the *P* < 0.05 level.

The results of the 2013 and 2014 field experiments suggested that healthy (not virus infected) plants were more susceptible to powdery mildew and that the transgene had no direct effect on susceptibility to powdery mildew. To explicitly test these findings, we conducted a greenhouse experiment that consisted of three treatments (mock-inoculated, virus-inoculated, and untreated controls) and used only wild gourds (no transgenic plants). A repeated measures ANOVA showed that treatment was a significant factor for predicting susceptibility to powdery mildew (*F*_2,287_ = 207.71, *P* < 0.001). We found that virus-infected plants showed significantly less severe powdery mildew symptoms than the other two treatments (mock-inoculated and control) and that mock-inoculated plants showed significantly more severe symptoms than the other two treatments (virus-infected and control) (**Figure 3**). There were significant family differences in powdery mildew severity as well (*F*_9,287_ = 118.56, *P* < 0.001) (**Supplementary Figure S4**), indicating broad sense heritable variation for powdery mildew resistance among the 10 families used in this study. A significantly smaller proportion of virus-infected plants were infected with powdery mildew relative to the mock-inoculated and control treatments at the early (*X*^2^ = 27.533, *df* = 2, *P* < 0.001) and middle (*X*^2^ = 6.582, *df* = 2, *P* = 0.037) time points (**Supplementary Table S2**). However, at the conclusion of the experiment, all plants were infected with powdery mildew.

### Phytohormone Analysis

**FIGURE 4:**
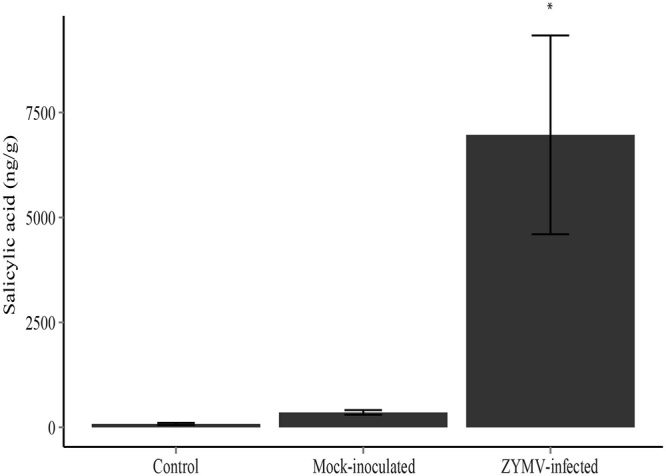
Mean salicylic acid levels with standard error for each treatment type (ZYMV-infected, control, and mock-inoculated) in the greenhouse experiment (*N* = 12 in each treatment). ZYMV-infected plants had significantly higher levels of salicylic acid (^∗^*P* < 0.05).

An ANOVA revealed significant differences in SA production among the treatments (mock-inoculated, virus-inoculated, and untreated controls) in our greenhouse study (*F*_2,33_ = 9.99, *P* < 0.001). A Tukey’s HSD test showed that leaves of ZYMV-infected plants contained significantly higher levels of SA compared to mock-inoculated and control plants (**Figure 4**).

### Effect of Powdery Mildew on Plant Fitness

A regression analysis revealed that there is a slight (but significant) negative impact on plant fitness as a result of powdery mildew infection in 2014 (**Supplementary Figure S5**). We did not conduct a regression analysis on the 2013 field data as there were only two powdery mildew scoring dates in 2013. However, an LS-means showed that susceptible plants produced 0.638 (*SE* = 0.012) of their total flower production and transgenic plants produced 0.628 (*SE* = 0.024) of their total flower production post first incidence of powdery mildew in our fields. We also used an analysis of covariance to assess the effects of weeks with powdery mildew (covariate), plant type (transgenic or susceptible), family (random), and treatment (inoculated or non-inoculated fields) on the proportion of both the total staminate and the total pistillate flowers produced by each plant after powdery mildew infection in both years. This analysis revealed that there was no difference in the proportion of flowers produced by transgenic and susceptible plants after powdery mildew infection in 2013 (Staminate: *F*_1,560_ = 0.03, *P* = 0.854, Pistillate: *F*_1,501_ = 0.53, *P* = 0.466). In contrast, our findings revealed that in 2014, transgenic plants produced a greater proportion of both staminate and pistillate flowers after powdery mildew infection relative to susceptible plants (Staminate: *F*_1,551_ = 10.29, *P* = 0.001; Pistillate: *F*_1,520_ = 6.97, *P* = 0.009). Moreover, even when weeks with virus is included as a second covariate in the model, transgenic plants still produce a significantly greater proportion of flowers than susceptible plants after powdery mildew infection (Staminate: *F*_1,551_ = 4.64, *P* = 0.032; Pistillate: *F*_1,520_ = 8.42, *P* = 0.004). This suggests that although powdery mildew infection has a negative impact on fitness, due to the timing of the diseases in our fields, the impact of powdery mildew was not as great as the impact of virus infection on plant fitness.

## Discussion

In multi-parasite systems, interactions among multiple diseases can lead to unexpected dynamics. In this study, we show that (1) plants infected with ZYMV are less likely to become infected with powdery mildew, (2) that when infected with powdery mildew, ZYMV-infected plants show reduced powdery mildew symptom severity, and (3) that infection with powdery mildew adversely affects the production of staminate and pistillate flowers. By August, when powdery mildew entered our ZYMV-inoculated fields, most non-virus-infected plants in our inoculated fields were transgenic individuals, however, our data show that the transgene has no direct effect on powdery-mildew infection (i.e., transgenic and susceptible plants were equally impacted by powdery mildew in the absence of ZYMV). Additionally, the greenhouse experiment (which did not use any transgenic plants) also showed that virus infected plants are more resistant to powdery mildew than their non-infected siblings, further indicating that the interaction between ZYMV and powdery mildew is not a direct result of transgene presence *per se*.

We also show that plants infected with ZYMV have elevated levels of SA post-infection (**Figure 4**), similar to previous work (Shapiro et al., 2013). These data, in combination with the field and greenhouse experiments in this study, suggest that a SAR response triggered by SA accumulation post-ZYMV infection increases the pathogen defenses of virus-infected plants compared to non-infected plants, thereby increasing their resistance to subsequent powdery-mildew infection. Other studies have shown that exposure to a pathogen can induce a SAR response that rarely results in complete resistance to a subsequent pathogen infection, but typically reduces lesion number and symptom severity (e.g., Kuc, 1982; Walters et al., 2005).

The mock-inoculated plants in our greenhouse study showed significantly more severe powdery mildew symptoms than both the control and virus-inoculated plants (**Figure 3**). While we expected that mock-inoculated plants would have more severe symptoms than the virus-inoculated plants, the fact that they had more severe symptoms than the control plants was surprising. The leaf damage caused by the inoculation/mock-inoculation procedure (abrasion) may have provided an easier avenue for the fungus to invade our plants. Additionally, the leaf damage may have upregulated the JA defense pathway, as commonly occurs with mechanical wounding, thereby suppressing levels of SA and facilitating powdery mildew infection as a result of antagonistic SA/JA cross-talk (Kunkel and Brooks, 2002; Chung et al., 2013). Regardless of the mechanism, the virus-inoculated plants received the same leaf abrasion as the mock inoculated plants, and still showed significantly less severe powdery mildew symptoms (**Figure 3**).

Previously, our lab documented an ecological, pre-infection interaction affecting exposure rates between two parasites in experimental populations of wild gourd. We found that in the absence of viral pathogens, virus-resistant transgenic and susceptible plants appear similarly affected by the deadly bacterial pathogen, *Erwinia tracheiphila*—the causative agent of bacterial wilt disease (Sasu et al., 2009). The disproportionate exposure of transgenic plants to wilt disease, due to avoidance of virus infected plants by vectors, mitigates the fitness advantage of the virus-resistant transgene when ZYMV is also epidemic in the population (Sasu et al., 2009, 2010a). Here, we present evidence of a second, post-infection interaction (a likely immunological interaction between ZYMV and powdery mildew) that further mitigates the benefit of the virus-resistant transgene. Our data reveal that powdery mildew adversely affects reproductive output and that ZYMV-infected plants are more resistant to powdery mildew than non-infected plants. During a viral outbreak, a high proportion of the non-infected plants possessed a virus-resistant transgene and suffered a greater incidence and severity of powdery mildew. Due to the influence of these pathogens on host-plant fitness, our findings have important implications for the spread of the transgene in natural populations of wild gourds.

Transgenic cultivars are often grown near wild populations and because gene flow between cultivated squash and wild gourds (*Cucurbita pepo* ssp. *texana*) is common and well documented (Kirkpatrick and Wilson, 1988; Decker-Walters et al., 2002), it is likely that the virus-resistant transgene has been introduced into wild populations. Unlike most traits of cultivated species which negatively influence fitness in wild populations, there are concerns that the presence of a virus-resistant transgene in wild populations could lead to increased fitness and weediness of wild species and/or have indirect impacts on non-target species and community composition (Fuchs and Gonsalves, 2007). Studies examining the transgene in both wild gourds and cultivated squash have shown that transgenic plants have a significant fitness advantage in the presence of the target pathogen and that there are no direct costs of transgene presence (Fuchs et al., 2004; Laughlin et al., 2009). In populations consisting of wild gourds and transgenic introgressives (as would happen if pollen flowed from transgenic cultivars into wild populations), the transgenic plants would suffer disproportionately from the adverse effects of powdery mildew if viral diseases are also present in the population. If all three diseases (ZYMV, bacterial wilt, and powdery mildew) are present in the same population, which seems likely, then fitness benefits of the transgene predicted by virus only studies would be greatly reduced. The pre-infection and post-infection interactions that would result from the presence of multiple pathogens in the same population are expected to slow, and under some conditions stop, the spread of the transgene in wild populations (Harth et al., 2012).

In addition to the implications for management of transgenic introgressives in wild populations, these data have implications for applied agriculture. Powdery mildew is a major economic pest of cultivated squashes and pumpkins worldwide by decreasing yield by as much as 50% and shortening the growing season (Perez-Garcia et al., 2009). Moreover, it is becoming increasingly difficult to control as some strains are resistant to the available fungicides (McGrath, 2001). Our data reveal heritable genetic variation for resistance to powdery mildew in wild *C. pepo* that could be bred into cultivated *C. pepo*. Moreover, recent studies have revealed that vertical transmission rates of ZYMV are 1.6% and that infected plants are virtually asymptomatic (Simmons et al., 2011). If vertically infected *C. pepo* show the same immune response to powdery mildew infection that horizontally infected ZYMV plants show in our studies, it could serve as a low pesticide alternative to extend the growing season. Similar cross-protection strategies in which a mild strain of one virus protects against a more virulent strain of that virus or another pathogen, have been effectively used in other crops (see reviews in Fulton, 1986; Lecoq and Raccah, 2001; Ziebell and Carr, 2010). For example, infection with the mild strain ZYMV-WK proved to be effective against infection with more severe ZYMV strains (Lecoq et al., 1991). Additionally, mild-strain cross protection using Citrus tristeza virus has successfully protected against more severe strains that cause citrus stem pitting (Folimonova, 2013) and has been important in maintaining profitability of the citrus market (Moreno et al., 2008). As in our study, it is likely that the mild pathogen induces defense pathways that in turn provide a degree of resistance to the more virulent pathogens (Lee and Keremane, 2013).

Plants in agricultural and especially natural populations are part of complex pathosystems consisting of a community of interacting organisms (Sasu et al., 2009) that have both direct and indirect effects on their host plants. Pairwise studies of hosts and their pathogens reveal only the direct effects of these pathogens; the direction and magnitude of indirect effects are far more difficult to predict. Studies of the *Cucurbita pepo* pathosystem reveal that a virus resistance transgene would spread rapidly through natural populations of wild gourd when only viral disease is present in the population. However, when multiple pathogens are present in the population, we see both pre-infection interactions and post-infection interactions among pathogens that would limit the spread of the transgene. The parallels between these mechanisms and those in animal systems suggest that these tractable plant systems might provide a test-bed for general tests of pathogen–pathogen–host interactions.

## Data Accessibility

The data supporting this manuscript are available via Penn State’s ScholarSphere and can be found at the following URL: https://scholarsphere.psu.edu/collections/4f16c298n.

## Author Contributions

JH, AS, and MF conceived and designed the experiments. JH performed the experiments. AH assisted with phytohormone experiments. JH, AS, AH, and MF analyzed the data. AS and JT contributed reagents, materials, and analysis tools. JH, AS, JT, and MF wrote the manuscript.

## Conflict of Interest Statement

The authors declare that the research was conducted in the absence of any commercial or financial relationships that could be construed as a potential conflict of interest.

## Supplementary Material

FIGURE S1 Schematic Representation of a 2013 Inoculated Field consisting of 15 rows and 12 columns of plants spaced evenly over a 0.4 ha plot, 180 total plants. Each box lists the type of plant (Wild type = X, non-transgenic backcross 9 = BC, transgenic backcross 9 = BCT) and the family to which it belongs (I2, J5, OZ, D2, or J3). Boxes shaded in red indicate ZYMV inoculated ntBC9 plants and boxes shaded in yellow indicate ZYMV inoculated wild type plants. The 2014 inoculated fields had the same layout.

FIGURE S2 Severity of powdery mildew on plants from each maternal family for each scoring date during the **(A)** 2013 and **(B)** 2014 field seasons. Different letters indicate significant differences between families at the *P* < 0.05 level.

FIGURE S3 Proportion of susceptible plants infected with powdery mildew in mid-August declines as the number of weeks for increasing weeks with zucchini yellow mosaic virus increases. Point size scales with number of plants that were virus infected for a given number of weeks.

FIGURE S4 Severity of powdery mildew symptoms for each maternal family included in the greenhouse experiment. Different letters indicate significant differences between groups at the *P* < 0.05 level.

FIGURE S5 Regression analysis for the impact of weeks infected with powdery mildew on the proportion of staminate (β = -0.0245, SE β = 0.0078, *R*^2^ = 1.72%, *P* = 0.002) and pistillate flowers (β = -0.07, SE β = 0.0121, *R*^2^ = 5.96%, *P* = 0.000) produced after first incidence of powdery mildew for 2014 field season. Dotted lines represent 95% confidence intervals.

TABLE S1 Percent of susceptible and transgenic plants infected with Zucchini yellow mosaic virus (ZYMV) and the percent of non-inoculated susceptible and transgenic plants infected with Powdery Mildew in both the ZYMV inoculated and not inoculated fields at each time point during the **(A)** 2013 field season and **(B)** 2014 field season.

TABLE S2 Percent of plants for each treatment type that showed powdery mildew at early and mid-time points during the greenhouse experiment.

## References

[B1] Alexander H. M. (2010). Disease in natural plant populations, communities, and ecosystems: insights into ecological and evolutionary processes. *Plant Dis.* 94 492–503. 10.1094/PDIS-94-5-0492 30754479

[B2] An C. Mou Z. (2011). Salicylic acid and its function in plant immunity. *J. Integr. Plant Biol.* 53 412–428. 10.1111/j.1744-7909.2011.01043.x 21535470

[B3] Anderson R. M. May R. M. (1978). Regulation and stability of host-parasite population interactions: I. Regulatory processes. *J. Anim. Ecol.* 47 219–247. 10.2307/3933

[B4] Arriaga L. Huerta E. Lira-Saade R. Moreno E. Alarcon J. (2006). Assessing the risk of releasing transgenic *Cucurbita* ssp. in Mexico. *Agric. Ecosyst. Environ.* 112 291–299. 10.1016/j.agee.2005.07.007

[B5] Burdon J. J. Leather S. R. (1990). *Pests, Pathogens and Plant Communities.* Oxford: Blackwell Scientific Publications.

[B6] Chung S. H. Rosa C. Scully E. D. Peiffer M. Tooker J. F. Hoover K. (2013). Herbivore exploits orally secreted bacteria to suppress plant defenses. *Proc. Natl. Acad. Sci. U.S.A.* 110 15728–15733. 10.1073/pnas.1308867110 24019469 PMC3785742

[B7] Cox F. E. G. (2001). Concomitant infections, parasites and immune responses. *Parasitology* 122 S23–S38. 10.1017/S003118200001698X 11442193

[B8] Dean R. A. Kuc J. (1987). Rapid lignification in response to wounding and infection as a mechanism for induced systemic protection in cucumber. *Physiol. Mol. Plant Pathol.* 31 69–81. 10.1016/0885-5765(87)90007-5

[B9] Decker-Walters D. S. Straub J. E. Chung S. M. Nakata E. Quemada H. D. (2002). Diversity in free-living populations of *Cucurbita pepo* (Cucurbitaceae) as assessed by random amplified polymorphic DNA. *Syst. Bot.* 27 19–28.

[B10] Decker-Walters D. S. Walters T. W. Cowan C. W. Smith B. D. (1993). Isozymic characterization of wild populations of *Cucurbita pepo*. *J. Ethnobiol.* 13 55–72.

[B11] Dobson R. J. Barnes E. H. (1995). Interaction between *Ostertagia circumcincta* and *Haemonchus contortus* infection in young lambs. *Int. J. Parasitol.* 25 495–501. 10.1016/0020-7519(94)00157-J 7635626

[B12] Donoghue H. D. Marcsik A. Matheson C. Vernon K. Nuorala E. Molto J. E. (2005). Co–infection of Mycobacterium tuberculosis and *Mycobacterium leprae* in human archaeological samples: a possible explanation for the historical decline of leprosy. *Proc. R. Soc. Lond. B Biol. Sci.* 272 389–394. 10.1098/rspb.2004.2966 15734693 PMC1634979

[B13] Du D. Winsor J. A. Smith M. DeNicco A. Stephenson A. G. (2008). Resistance and tolerance to herbivory changes with inbreeding and ontogeny in a wild gourd (Cucurbitaceae). *Am. J. Bot.* 95 84–92. 10.3732/ajb.95.1.84 21632318

[B14] Ferrari M. Winsor J. A. Du D. Stephenson A. G. (2007). Inbreeding alters host plant quality and incidence of an insect borne pathogen in *Cucurbita pepo* ssp. *texana*. *Int. J. Plant Sci.* 168 603–610. 10.1086/513487

[B15] Fleischer S. J. de Mackiewicz D. Gildow F. E. Lukezic F. L. (1999). Serological estimates of the seasonal dynamics of *Erwinia tracheiphila* in *Acalymma vittata* (Coleoptera: Chrysomelidae). *Environ. Entomol.* 28 470–476. 10.1093/ee/28.3.470

[B16] Folimonova S. Y. (2013). Developing an understanding of cross-protection by *Citrus tristeza virus*. *Front. Microbiol.* 4:76. 10.3389/fmicb.2013.00076 23577008 PMC3616238

[B17] Fuchs M. Chirco E. M. McFerson J. M. Gonsalves D. (2004). Comparative fitness of a wild squash species and three generations of hybrids between wild X virus-resistant transgenic squash. *Enivron. Biosaf. Res.* 3 17–28. 10.1051/ebr:2004004 15612352

[B18] Fuchs M. Gonsalves D. (2007). Safety of virus-resistant transgenic plants two decades after their introduction: lessons from realistic field risk assessment studies. *Annu. Rev. Phytopathol.* 45 173–202. 10.1146/annurev.phyto.45.062806.094434 17408355

[B19] Fulton R. W. (1986). Practices and precautions in the use of cross protection for plant virus disease control. *Annu. Rev. Phytopathol.* 24 67–81. 10.1146/annurev.py.24.090186.000435

[B20] Harth J. E. Deveny T. W. Winsor J. A. Ferrari M. A. Stephenson A. G. (2012). “Background and development of a model to predict the fate of an escaped virus resistant transgene from *Cucurbita pepo*,” in *Proceedings of the 10th EUCARPIA Meeting on Genetics and Breeding of Cucurbitaceae 2012*, eds Sari N. Solmaz I. Aras V. (Antalya: University of Cukurova).

[B21] Harth J. E. Winsor J. A. Weakland D. R. Nowak K. J. Ferrari M. J. Stephenson A. G. (2016). Effects of virus infection on pollen production and pollen performance: implications for the spread of resistance alleles. *Am. J. Bot.* 103 577–583. 10.3732/ajb.1500165 26905087

[B22] Hatcher M. J. Dick J. T. Dunn A. M. (2006). How parasites affect interactions between competitors and predators. *Ecol. Lett.* 9 1253–1271. 10.1111/j.1461-0248.2006.00964.x 17040328

[B23] Hawkins B. A. Cornell H. V. Hochberg M. E. (1997). Predators, parasitoids, and pathogens as mortality agents in phytophagous insect populations. *Ecology* 78 2145–2152. 10.1890/0012-9658(1997)078[2145:PPAPAM]2.0.CO;2

[B24] Hayes C. N. Winsor J. A. Stephenson A. G. (2004). Inbreeding influences herbivory in *Cucurbita pepo* ssp. *texana* (Cucurbitaceae). *Oecologia* 140 601–608. 10.1007/s00442-004-1623-2 15252728

[B25] Ishii T. Takatsuka J. Nakai M. Kunimi Y. (2002). Growth characteristics and competitive abilities of a nucleopolyhedrovirus and an entomopoxvirus in larvae of the smaller tea tortrix, *Adoxophyes honmai* (Lepidoptera: Tortricidae). *Biol. Control* 23 96–105. 10.1006/bcon.2001.0988

[B26] Jones J. D. Dangl J. L. (2006). The plant immune system. *Nature* 444 323–329. 10.1038/nature05286 17108957

[B27] Kirkpatrick K. J. Wilson H. D. (1988). Interspecific gene flow in *Cucurbita*: *C. texana* vs. *C. pepo*. *Am. J. Bot.* 75 519–527. 10.1002/j.1537-2197.1988.tb13470.x

[B28] Kuc J. (1982). Induced immunity to plant disease. *Bioscience* 32 854–860. 10.2307/1309008

[B29] Kunkel B. N. Brooks D. M. (2002). Cross talk between signaling pathways in pathogen defense. *Curr. Opin. Plant Biol.* 5 325–331. 10.1016/S1369-5266(02)00275-3 12179966

[B30] Laughlin K. D. Power A. G. Snow A. A. Spencer L. J. (2009). Risk Assesment of genetically engineered crops: fitness effects of virus-resistance transgenes in wild *Cucurbita pepo*. *Ecol. Appl.* 19 1091–1101. 10.1890/08-0105.1 19688918

[B31] Lecoq H. Lemaire J. M. Wipf-Scheibel C. (1991). Control of zucchini yellow mosaic virus in squash by cross protection. *Plant Dis.* 75 208–211. 10.1094/PD-75-0208

[B32] Lecoq H. Raccah B. (2001). “Cross-protection: interactions between strains exploited to control plant virus diseases,” in *Biotic Interactions in Plant-Pathogen Associations*, eds Jegger M. J. Spence N. J. (Wallingford: CAB International), 177 10.1079/9780851995120.0177

[B33] Lee R. F. Keremane M. L. (2013). Mild strain cross protection of tristeza: a review of research to protect against decline on sour orange in Florida. *Front. Microbiol.* 4:39–49. 10.3389/fmicb.2013.00259 24046764 PMC3764332

[B34] Lello J. Boag B. Fenton A. Stevenson I. R. Hudson P. J. (2004). Competition and mutualism among the gut helminths of a mammalian host. *Nature* 428 840–844. 10.1038/nature02490 15103373

[B35] Lively C. M. de Roode J. C. Duffy M. A. Graham A. L. Koskella B. (2014). Interesting open questions in disease ecology and evolution. *Am. Nat.* 184 1–8. 10.1086/677032 25061674

[B36] Maskell L. C. Raybould A. F. Cooper J. I. Edwards M. L. Gray A. J. (1999). Effects of turnip mosaic virus and turnip yellow mosaic virus on the survival, growth and reproduction of wild cabbage (Brassica oleracea). *Ann. Appl. Biol.* 135 401–407. 10.1111/j.1744-7348.1999.tb00867.x

[B37] Mauck K. E. De Moraes C. M. Mescher M. C. (2010). Deceptive chemical signals induced by a plant virus attract insect vectors to inferior hosts. *Proc. Natl. Acad. Sci. U.S.A.* 107 3600–3605. 10.1073/pnas.0907191107 20133719 PMC2840436

[B38] McGrath M. T. (2001). Fungicide resistance in cucurbit powdery mildew: experiences and challenges. *Plant Dis.* 85 236–245. 10.1094/PDIS.2001.85.3.236 30832035

[B39] Mitchell C. E. Power A. D. (2006). “Disease dynamics in plant communities,” in *Disease Ecology: Community Structure and Pathogen Dynamics*, eds Collinge S. K. Ray C. (Oxford: Oxford University Press).

[B40] Moore J. (2002a). *Parasites and the Behavior of Animals.* Oxford: Oxford University Press.

[B41] Moore J. (2002b). Host behavioral manipulation. *Trends Parasitol.* 18:10.

[B42] Moreno P. Ambros S. Albiach-Marti M. R. Guerri J. Pena J. (2008). *Citrus tristeza virus*: a pathogen that changed the course of the citrus industry. *Mol. Plant Pathol.* 9 251–268. 10.1111/j.1364-3703.2007.00455.x 18705856 PMC6640355

[B43] Mucharromah E. Kuc J. (1991). Oxalate and phosphates induce systemic resistance against diseases caused by fungi, bacteria and viruses in cucumber. *Crop Prot.* 10 265–270. 10.1016/0261-2194(91)90004-B

[B44] Pedersen A. B. Fenton A. (2006). Emphasizing the ecology in parasite community ecology. *Trends Ecol. Evol.* 22 133–139. 10.1016/j.tree.2006.11.005 17137676

[B45] Perez-Garcia A. Romero D. Fernandez-Ortuno D. Lopez-Ruiz F. De Vicente A. Tores J. A. (2009). The powdery mildew fungus *Podosphaera fusca* (synonym *Podosphaera xanthii*), a constant threat to cucurbits. *Mol. Plant Pathol.* 153–160. 10.1111/j.1364-3703.2008.00527.x 19236565 PMC6640438

[B46] Petney T. N. Andrews R. H. (1998). Multiparasite communities in animals and humans: frequency, structure and pathogenic significance. *Int. J. Parasitol.* 28 377–393. 10.1016/S0020-7519(97)00189-6 9559357

[B47] Prendeville H. R. Ye X. Morris T. J. Pilson D. (2012). Virus infections in wild plant populations are both frequent and often unapparent. *Am. J. Bot.* 99 1033–1042. 10.3732/ajb.1100509 22645099

[B48] Rohani P. Earn D. J. Finkenstadt B. Grenfell B. T. (1998). Population dynamic interference among childhood diseases. *Proc. R. Soc. Lond. B* 265 2033–2041. 10.1098/rspb.1998.0537 9842732 PMC1689490

[B49] Rohani P. Green C. J. Mantilla-Beniers N. B. Grenfell B. T. (2003). Ecological interference between fatal diseases. *Nature* 422 885–888. 10.1038/nature01542 12712203

[B50] Ryals J. A. Neuenschwander U. H. Willits M. G. Molina A. Steiner H. Y. Hunt M. D. (1996). Systemic acquired resistance. *Plant Cell* 8 1809–1819. 10.1105/tpc.8.10.1809 12239363 PMC161316

[B51] Sasu M. A. Ferrari M. J. Du D. Winsor J. A. Stephenson A. G. (2009). Indirect costs of a non-target pathogen mitigate the direct benefits of a virus-resistant transgene in wild *Cucurbita*. *Proc. Natl. Acad. Sci. U.S.A.* 106 19067–19071. 10.1073/pnas.0905106106 19858473 PMC2776422

[B52] Sasu M. A. Ferrari M. J. Stephenson A. G. (2010a). Interrelationships among a virus-resistant transgene, herbivory, and a bacterial disease in wild *Cucurbita*. *Int. J. Plant Sci.* 171 1048–1058.

[B53] Sasu M. A. Seidl-Adams I. Wall K. Winsor J. A. Stephenson A. G. (2010b). Floral transmission of *Erwinia tracheiphila* by cucumber beetles in a wild *Cucurbita pepo*. *Environ. Entomol.* 39 140–148. 10.1603/EN09190 20146850

[B54] Schmelz E. A. Engelberth J. Alborn H. T. O’Donnell P. Sammons M. Toshima H. (2003). Simultaneous analysis of phytohormones, phytotoxins, and volatile organic compounds in plants. *Proc. Natl. Acad. Sci. U.S.A.* 100 10552–10557. 10.1073/pnas.1633615100 12874387 PMC193599

[B55] Schmelz E. A. Engelberth J. Tumlinson J. H. Block A. Alborn H. T. (2004). The use of vapor phase extraction in metabolic profiling of phytohormones and other metabolites. *Plant J.* 39 790–808. 10.1111/j.1365-313X.2004.02168.x 15315639

[B56] Shapiro L. Moraes C. M. Stephenson A. G. Mescher M. C. (2012). Pathogen effects on vegetative and floral odours mediate vector attraction and host exposure in a complex pathosystem. *Ecol. Lett.* 15 1430–1438. 10.1111/ele.12001 22988893

[B57] Shapiro L. R. Salvaudon L. Mauck K. E. Pulido H. De Moraes C. M. Stephenson A. G. (2013). Disease interactions in a shared host plant: effects of pre-existing viral infection on cucurbit plant defense responses and resistance to bacterial wilt disease. *PLoS One* 8:e77393. 10.1371/journal.pone.0077393 24155951 PMC3796458

[B58] Shapiro L. R. Seidl-Adams I. De Moraes C. M. Stephenson A. G. Mescher M. C. (2014). Dynamics of short-and long-term association between a bacterial plant pathogen and its arthropod vector. *Sci. Rep.* 4:4155. 10.1038/srep04155 24561664 PMC3932477

[B59] Simmons H. E. Holmes E. C. Gildow F. E. Bothe-Goralcyzk M. A. Stephenson A. G. (2011). Experimental verification of seed transmission of *Zucchini yellow mosaic virus*. *Plant Dis.* 95 751–754. 10.1094/PDIS-11-10-0843 30731907

[B60] Stephenson A. G. Leyson B. Travers S. E. Hayes C. N. Winsor J. A. (2004). Interrelationships among inbreeding, herbivory, and disease on reproduction in a wild gourd. *Ecology* 85 3023–3034. 10.1890/04-0005

[B61] Stewart G. R. Boussinesq M. Coulson T. Elson L. Nutman T. Bradley J. E. (1999). Onchocerciasis modulates the immune response to mycobacterial antigens. *Clin. Exp. Immunol.* 117 517–523. 10.1046/j.1365-2249.1999.01015.x 10469056 PMC1905356

[B62] Strauss S. Y. (1991). Indirect effects in community ecology: their definition, study and importance. *Trends Ecol. Evol.* 6 206–210. 10.1016/0169-5347(91)90023-Q 21232460

[B63] USDA (1996). *Environmental Assessment for Upjohn Company/Asgrow Seed Company Petition for Determination of Non-Regulated Status for CZW-3 Squash.* Washington, DC: US Department of Agriculture.

[B64] Vlot A. C. Dempsey D. M. A. Klessig D. F. (2009). Salicylic acid, a multifaceted hormone to combat disease. *Annu. Rev. Phytopathol.* 47 177–206. 10.1146/annurev.phyto.050908.135202 19400653

[B65] Walters D. Walsh D. Newton A. Lyon G. (2005). Induced resistance for plant disease control: maximizing the efficacy of resistance elicitors. *Phytopathology* 95 1368–1373. 10.1094/PHYTO-95-1368 18943546

[B66] Wang H. Xu D. Pu L. Zhou G. (2014). Southern rice black-streaked dwarf virus alters insect vectors’ host orientation preferences to enhance spread and increase rice ragged stunt virus co-infection. *Phytopathology* 104 196–201. 10.1094/PHYTO-08-13-0227-R 24047253

[B67] Wasternack C. (2007). Jasmonates: an update on biosynthesis, signal transduction and action in plant stress response, growth and development. *Ann. Bot.* 100 681–697. 10.1093/aob/mcm079 17513307 PMC2749622

[B68] Ziebell H. Carr J. P. (2010). Cross-protection: a century of mystery. *Adv. Virus Res.* 76 211–264. 10.1016/S0065-3527(10)76006-1 20965075

