# Corrigendum: Zucchini Yellow Mosaic Virus Infection Limits Establishment and Severity of Powdery Mildew in Wild Populations of *Cucurbita pepo*

**DOI:** 10.3389/fpls.2018.01815

**Published:** 2018-12-10

**Authors:** Jacquelyn E. Harth, Matthew J. Ferrari, Anjel M. Helms, John F. Tooker, Andrew G. Stephenson

**Affiliations:** ^1^Department of Biology, The Pennsylvania State University, University Park, PA, United States; ^2^Center for Infectious Disease Dynamics, The Pennsylvania State University, University Park, PA, United States; ^3^Department of Entomology, The Pennsylvania State University, University Park, PA, United States

**Keywords:** *Cucurbita pepo*, multi-parasite interactions, powdery mildew, systemic acquired resistance, ZYMV

Anjel M. Helms was not included as an author in the published article. The authors apologize for this error and state that this does not change the scientific conclusions of the article in any way. The original article has been updated.

## Conflict of Interest Statement

The authors declare that the research was conducted in the absence of any commercial or financial relationships that could be construed as a potential conflict of interest.

