# Supplementary material for: Zucchini Yellow Mosaic Virus Infection Limits Establishment and Severity of Powdery Mildew in Wild Populations of Cucurbita pepo
Source: Front Plant Sci. 2018 Jun 13;9:792. doi: 10.3389/fpls.2018.00792 (PMC6008421; doi:10.3389/fpls.2018.00792)
Supplement: FIGURE S1 — Schematic Representation of a 2013 Inoculated Field consisting of 15 rows and 12 columns of plants spaced evenly over a 0.4 ha plot, 180 total plants. Each box lists the type of plant (Wild type = X, non-transgenic backcross 9 = BC, transgenic backcross 9 = BCT) and the family to which it belongs (I2, J5, OZ, D2, or J3). Boxes shaded in red indicate ZYMV inoculated ntBC9 plants and boxes shaded in yellow indicate ZYMV inoculated wild type plants. The 2014 inoculated fields had the same layout. [file Image_1.PDF]

	A	B	C	D	E	F	G	H	I	J	K	L
1	I2-X	BC T I2	J5-X	BC I2	OZ-X	BC T I2	J3-X	BC I2	D2-X	BC T I2	I2-X	BC I2
2	BC TOZ	J5-X	BC OZ	OZ-X	BC TOZ	J3-X	BC OZ	D2-X	BC T OZ	I2-X	BC OZ	J5-X
3	OZ-X	BC D2	J3-X	BC T D2	D2-X	BC D2	D2-X	BC T D2	OZ-X	BC D2	J3-X	BC T D2
4	BC J3	J3-X	BC T J3	OZ-X	BC J3	J5-X	BC T J3	I2X	BC J3	J5-X	BC T J3	I2X
5	D2-X	BC T J5	D2-X	BC J5	J3-X	BC T J5	OZ-X	BC J5	J5-X	BC T J5	I2-X	BC J5
6	BC T I2	J3-X	BC I2	D2-X	BC T I2	D2-X	BC I2	I2-X	BC T I2	J5-X	BC I2	OZ-X
7	I2-X	BC OZ	J5-X	BC T OZ	OZ-X	BC OZ	J3-X	BC T OZ	I2-X	BC OZ	J5-X	BC T OZ
8	BC D2	D2-X	BC T D2	OZ-X	BC D2	J3-X	BC T D2	J3-X	BC D2	OZ-X	BC T D2	D2-X
9	J5-X	BC T J3	I2X	BC J3	J5-X	BC T J3	I2X	BC J3	J3-X	BC T J3	OZ-X	BC J3
10	BC T J5	OZ-X	BC J5	J5-X	BC T J5	I2-X	BC J5	D2-X	BC T J5	D2-X	BC J5	J3-X
11	D2-X	BC I2	I2-X	BC T I2	J5-X	BC I2	OZ-X	BC T I2	J3-X	BC I2	D2-X	BC T I2
12	BC OZ	J3-X	BC T OZ	I2-X	BC OZ	J5-X	BC T OZ	I2-X	BC OZ	J5-X	BC T OZ	OZ-X
13	J3-X	BC T D2	J3-X	BC D2	OZ-X	BC T D2	D2-X	BC D2	D2-X	BC T D2	OZ-X	BC D2
14	BC T J3	I2X	BC J3	J3-X	BC T J3	OZ-X	BC J3	J5-X	BC T J3	I2X	BC J3	J5-X
15	I2-X	BC J5	D2-X	BC T J5	D2-X	BC J5	J3-X	BC T J5	OZ-X	BC J5	J5-X	BC T J5
