# Supplementary figures and images for: Zucchini Yellow Mosaic Virus Infection Limits Establishment and Severity of Powdery Mildew in Wild Populations of Cucurbita pepo
Source: Front Plant Sci. 2018 Jun 13;9:792. doi: 10.3389/fpls.2018.00792 (PMC6008421; doi:10.3389/fpls.2018.00792)

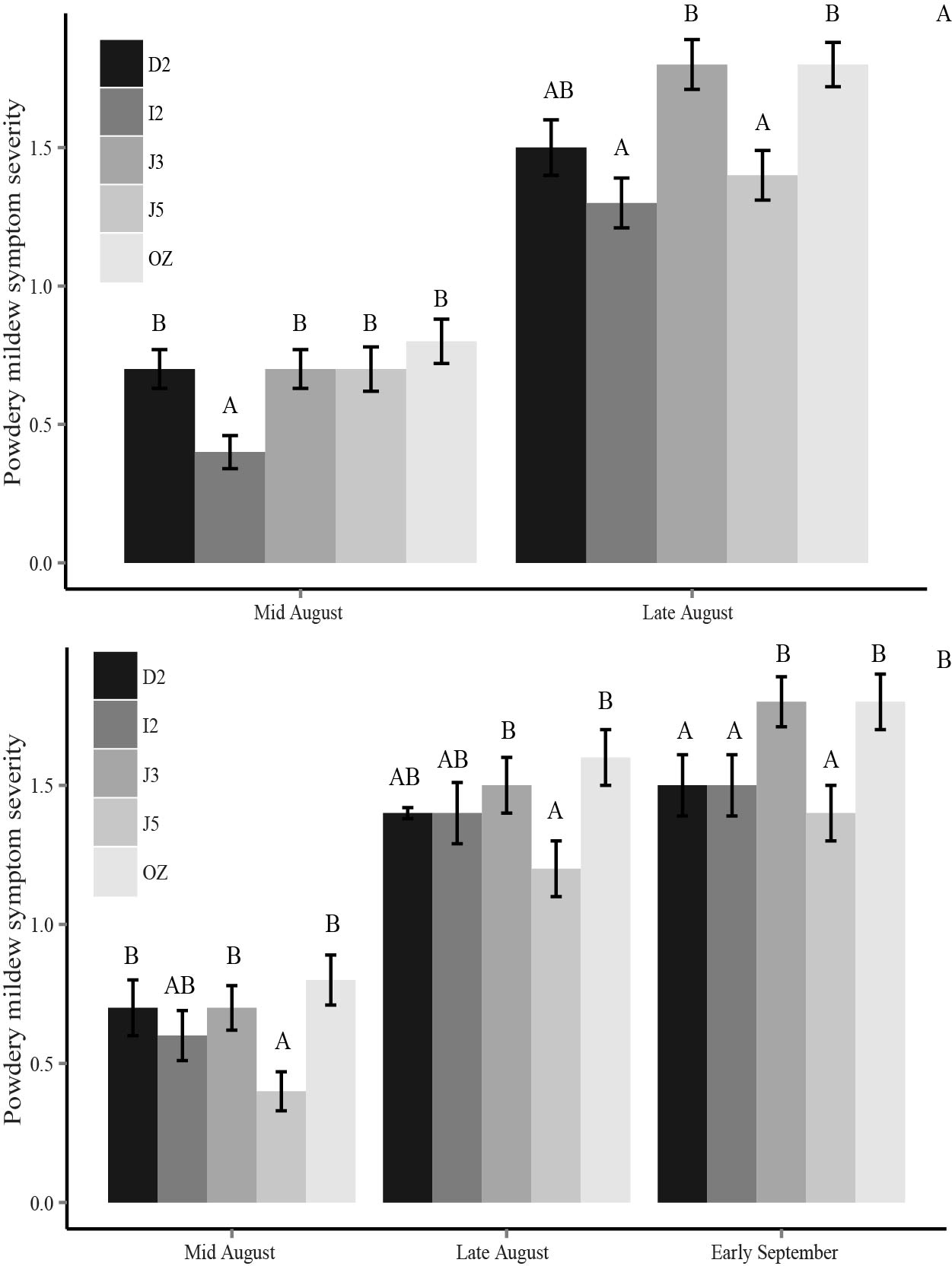

Supplement: FIGURE S2 — Severity of powdery mildew on plants from each maternal family for each scoring date during the (A) 2013 and (B) 2014 field seasons. Different letters indicate significant differences between families at the P < 0.05 level. [file Image_2.JPEG]

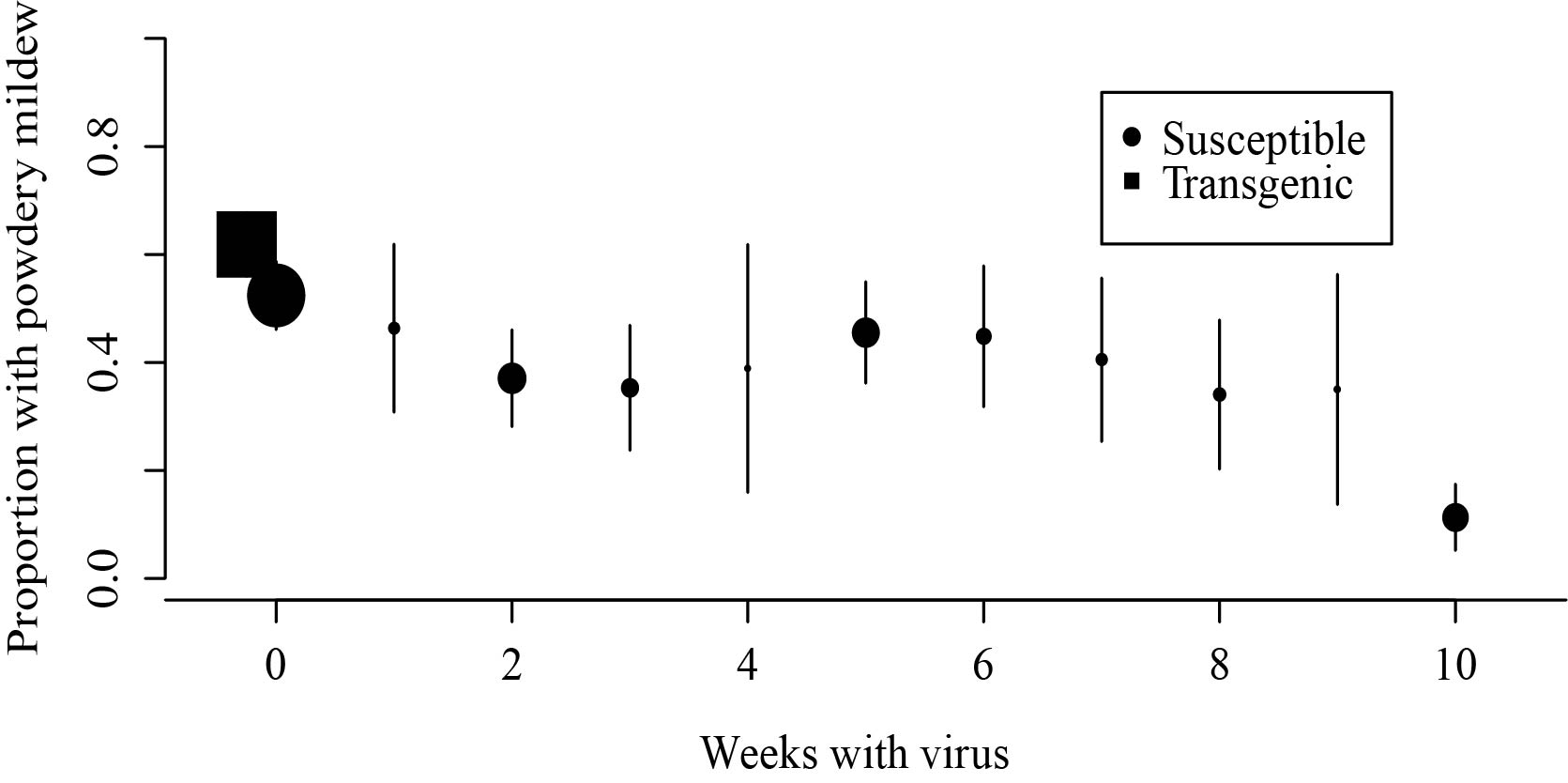

Supplement: FIGURE S3 — Proportion of susceptible plants infected with powdery mildew in mid-August declines as the number of weeks for increasing weeks with zucchini yellow mosaic virus increases. Point size scales with number of plants that were virus infected for a given number of weeks. [file Image_3.jpg]

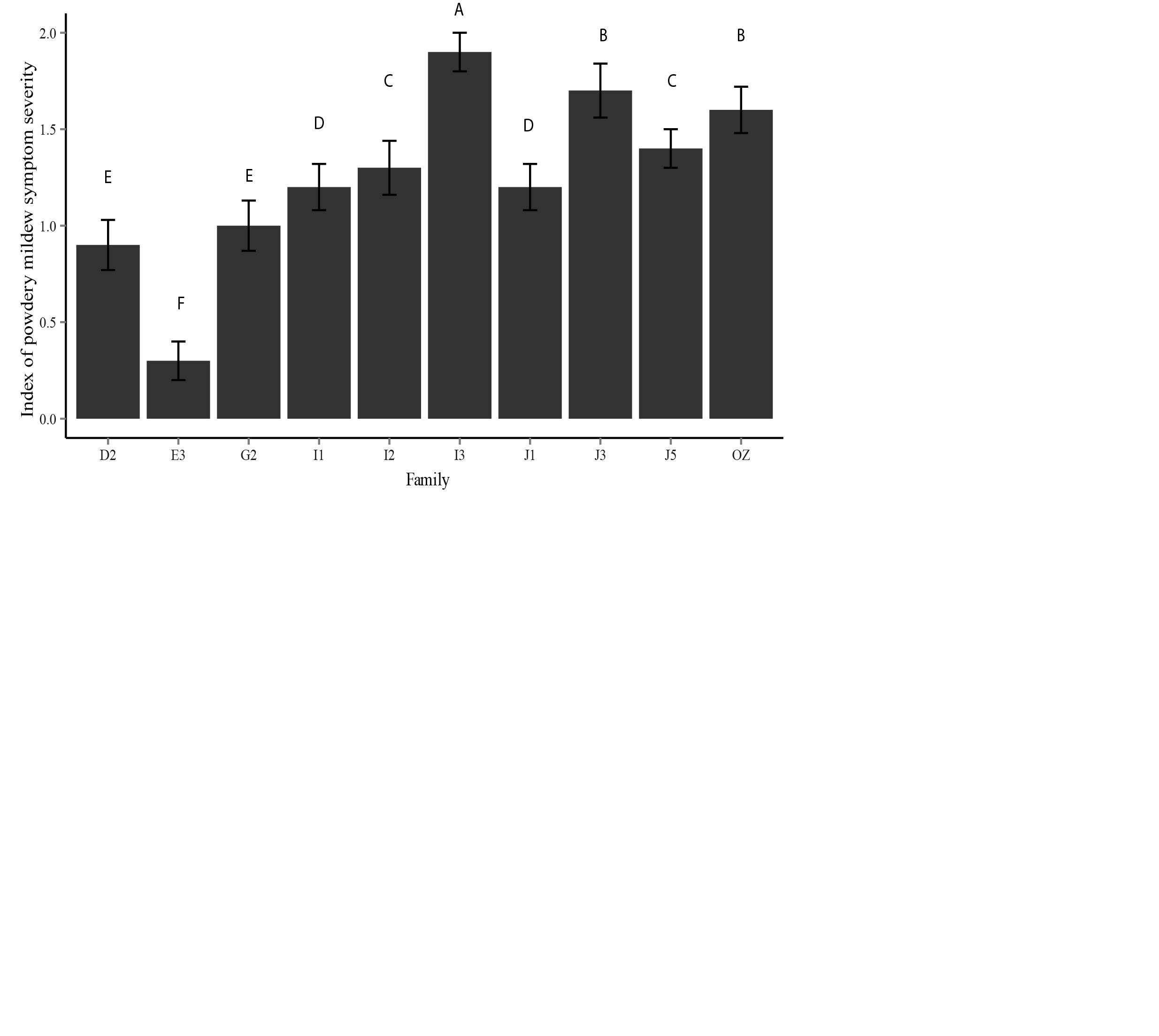

Supplement: FIGURE S4 — Severity of powdery mildew symptoms for each maternal family included in the greenhouse experiment. Different letters indicate significant differences between groups at the P < 0.05 level. [file Image_4.JPEG]

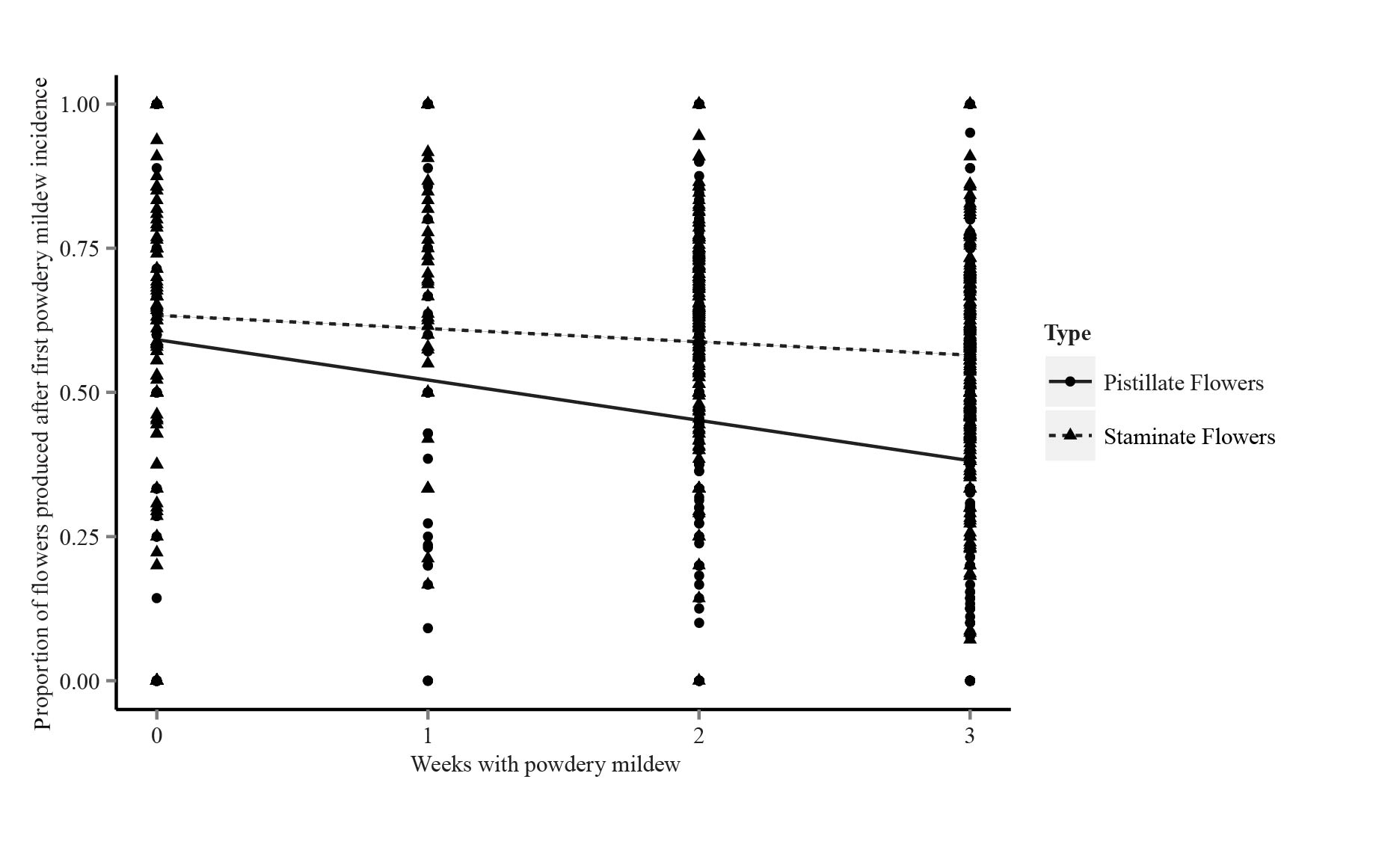

Supplement: FIGURE S5 — Regression analysis for the impact of weeks infected with powdery mildew on the proportion of staminate (β = -0.0245, SE β = 0.0078, R2 = 1.72%, P = 0.002) and pistillate flowers (β = -0.07, SE β = 0.0121, R2 = 5.96%, P = 0.000) produced after first incidence of powdery mildew for 2014 field season. Dotted lines represent 95% confidence intervals. [file Image_5.JPEG]
