# Supplementary material for: Zucchini Yellow Mosaic Virus Infection Limits Establishment and Severity of Powdery Mildew in Wild Populations of Cucurbita pepo
Source: Front Plant Sci. 2018 Jun 13;9:792. doi: 10.3389/fpls.2018.00792 (PMC6008421; doi:10.3389/fpls.2018.00792)
Supplement: TABLE S1 — Percent of susceptible and transgenic plants infected with Zucchini yellow mosaic virus (ZYMV) and the percent of non-inoculated susceptible and transgenic plants infected with Powdery Mildew in both the ZYMV inoculated and not inoculated fields at each time point during the (A) 2013 field season and (B) 2014 field season. [file Table_1.DOCX]

Supplemental Table 1. Percent of susceptible and transgenic plants infected with Zucchini yellow mosaic virus (ZYMV) and the percent of non-inoculated susceptible and transgenic plants infected with Powdery Mildew in both the ZYMV inoculated and not inoculated fields at each time point during the A) 2013 field season and B) 2014 field season.

| A | Inoculated Fields | | | | Not Inoculated Fields | | | |
| --- | --- | --- | --- | --- | --- | --- | --- | --- |
|  | ZYMV Infected | | Powdery Mildew Infected | | ZYMV Infected | | Powdery Mildew Infected | |
| 2013 | Transgenic | Susceptible | Transgenic | Susceptible | Transgenic | Susceptible | Transgenic | Susceptible |
| July 2^nd^ | 0% | 24.9% | 0% | 0% | 0% | 0% | 0% | 0% |
| July 9^th^ | 0% | 31.6% | 0% | 0% | 0% | 0% | 0% | 0% |
| July 16^th^ | 0% | 46.4% | 0% | 0% | 0% | 0% | 0% | 0% |
| July 23^rd^ | 0% | 59.5% | 0% | 0% | 0% | 0% | 0% | 0% |
| July 30^st^ | 0% | 77.1% | 0% | 0% | 0% | 0% | 0% | 0% |
| August 6^th^ | 0% | 79.4% | 0% | 0% | 0% | 0.8% | 0% | 0% |
| August 13^th^ | 0% | 82.5% | 0% | 0% | 0% | 0.8% | 0% | 0% |
| August 20^th^ | 1.3% | 87.1% | 56.4% | 28.6% | 0% | 1.7% | 76.3% | 54.0% |
| August 27^th^ | 2.6% | 89.9% | 83.3% | 67.5% | 0% | 8.3% | 86.7% | 63.3% |
| September 3^rd^ | 2.7% | 92.6% | 95.9% | 87.0% | 2.7% | 20.4% | 90.5% | 75.6% |

| B | Inoculated Fields | | | | Not Inoculated Fields | | | |
| --- | --- | --- | --- | --- | --- | --- | --- | --- |
|  | ZYMV Infected | | Powdery Mildew Infected | | ZYMV Infected | | Powdery Mildew Infected | |
| 2014 | Transgenic | Susceptible | Transgenic | Susceptible | Transgenic | Susceptible | Transgenic | Susceptible |
| July 2^nd^ | 0% | 17.4% | 0% | 0% | 0% | 0% | 0% | 0% |
| July 9^th^ | 0% | 18.0% | 0% | 0% | 0% | 0% | 0% | 0% |
| July 17^th^ | 0% | 22.5% | 0% | 0% | 0% | 0% | 0% | 0% |
| July 23^rd^ | 0% | 27.2% | 0% | 0% | 0% | 0% | 0% | 0% |
| July 31^st^ | 0% | 33.9% | 0% | 0% | 0% | 0% | 0% | 0% |
| August 7^th^ | 0% | 84.7% | 0% | 0% | 0% | 0% | 0% | 0% |
| August 13^th^ | 0% | 85.6% | 50% | 26.8% | 0% | 0% | 16.4% | 19.6% |
| August 20^th^ | 1.4% | 89.8% | 66.7% | 40.4% | 1.4% | 18.4% | 36.6% | 33.6% |
| August 27^th^ | 1.5% | 86.8% | 85.9% | 75.9% | 1.5% | 61.2% | 75.4% | 63.7% |
| September 3^rd^ | 2.9% | 87.2% | 95.5% | 88.6% | 2.9% | 66.4% | 85.5% | 69.8% |
