# Supplementary material for: Zucchini Yellow Mosaic Virus Infection Limits Establishment and Severity of Powdery Mildew in Wild Populations of Cucurbita pepo
Source: Front Plant Sci. 2018 Jun 13;9:792. doi: 10.3389/fpls.2018.00792 (PMC6008421; doi:10.3389/fpls.2018.00792)
Supplement: TABLE S2 — Percent of plants for each treatment type that showed powdery mildew at early and mid-time points during the greenhouse experiment. [file Table_2.DOCX]

Supplemental Table 2. Percent of plants for each treatment type that showed powdery mildew at early and mid-time points during the greenhouse experiment.

| Powdery Mildew? |  | Yes | No |
| --- | --- | --- | --- |
|  |  |  |  |
| *Early* | Control | 39.5% | 61.5% |
|  | Mock | 44.4% | 56.6% |
|  | Virus | 15.3% | 84.7% |
|  |  |  |  |
| *Middle* | Control | 84.5% | 14.5% |
|  | Mock | 90.1% | 9.9% |
|  | Virus | 77.0% | 23.0% |
